# Intraocular Inflammation Secondary to Intravitreal Brolucizumab Injection for Neovascular Age-Related Macular Degeneration in a Patient with Cognitive Impairment

**DOI:** 10.3390/medicina59101856

**Published:** 2023-10-19

**Authors:** Natsuki Ueji, Yoko Mase, Akiko Kubo, Hisashi Matsubara, Shinichiro Chujo, Yoshitsugu Matsui, Mineo Kondo

**Affiliations:** 1 Ophthalmology, Kinan Hospital, 4750 Atawa, Mihama-cho, Minaminuro-gun 519-5293, Japan; yokosun9@gmail.com (Y.M.); akubo@mxj.mesh.ne.jp (A.K.); 2 Department of Ophthalmology, Mie University Graduate School of Medicine, 2-174 Edobashi, Tsu-shi 514-8507, Japan; hmatsu@med.mie-u.ac.jp (H.M.); shin21@med.mie-u.ac.jp (S.C.); footboyslim366@gmail.com (Y.M.); mineo@med.mie-u.ac.jp (M.K.)

**Keywords:** age-related macular degeneration, AMD, intraocular inflammation, IOI, brolucizumab, dementia, cognitive impairment

## Abstract

*Background and Objectives*: Brolucizumab (IVBr) is a recently introduced anti-vascular endothelial growth factor (anti-VEGF) which has been found to be very effective in treating neovascular age-related macular degeneration (nAMD). We reported our findings in a case of nAMD that developed intraocular inflammation (IOI) after IVBr injections. *Materials and Methods*: A 79-year-old man was referred to our hospital complaining of reduced vision in both eyes of one-month’s duration. His decimal best-corrected visual acuity (BCVA) was 0.9 in the right eye and 1.0 in the left eye. He was diagnosed with nAMD in the left eye and was treated with intravitreal aflibercept (IVA). Despite the three-monthly IVA injections, the serous retinal pigment epithelial detachment (PED) and subretinal fluid (SRF) remained, and the VA gradually decreased to 0.1. Because of the patient being refractory to aflibercept treatment, we switched to 3-monthly IVBr injections. The BCVA gradually improved to 0.3 and optical coherence tomography (OCT) showed an absence of the serous PED and SRF. Three weeks after his third IVBr, he returned to our hospital with a complaint of reduced vision in his left eye that he first noted two weeks earlier. Our examination of the left eye showed signs of IOI mainly in the anterior chamber. The inflammation improved with topical steroids but the treatment of the IOI was delayed for two weeks. The patient was instructed that it was important to begin the treatment as soon as the symptoms of IOI developed. We then performed the Mini-Mental State Examination (MMSE), and his score indicated that he had cognitive impairment. *Conclusions*: We concluded that before beginning IVBr treatment in nAMD patients, a careful assessment must be made of the cognitive status of the patient.

## 1. Introduction

It has been well documented that intravitreal injections of anti-vascular endothelial growth factor (anti-VEGF) drugs lead to functional and anatomical improvements of eyes with neovascular age-related macular degeneration (nAMD). Because of their effectiveness, anti-VEGF treatments have become the first-line treatment for nAMD; however, the treatment has several weaknesses including its lack of effect on exudations and the need for frequent intravitreal injections.

Thus, new anti-VEGF drugs have been developed that have stronger effects on exudations and longer-lasting effects. One of these newly developed anti-VEGF agents is brolucizumab (Beovu; Novartis Pharma AG, Basel, Switzerland) which is a humanized single-chain antibody fragment that inhibits the binding of VEGF-A to the VEGF receptor. It was approved for use in Japan in 2020. Its lower molecular weight compared to other commercially available anti-VEGF drugs allows it to be used at a higher molar concentration which then has greater therapeutic effects. However, several post-marketing cases of visual acuity reduction caused by severe intraocular inflammation (IOI) and retinal vasculitis and/or retinal artery occlusion (RAO) have been reported after intravitreal injection of brolucizumab (IVBr) [1]. The Safety Review Committee (SRC) reported that IOI in any form was identified in 50 of 1088 study eyes of patients treated with brolucizumab (4.6%) during the HAWK and HARRIER trials. Of these 50 eyes with IOI, 36 eyes had concomitant retinal vasculitis (3.3%), of which 23 had concomitant retinal vascular occlusion (2.1%) [2]. These studies reported that an early diagnosis and appropriate treatments were necessary to treat the IOI successfully. Because the detection of IOI is usually made by complaints from patients of a worsening of the vision, it is imperative that patients know the symptoms associated with IOI. Once the diagnosis of IOI is confirmed, initiation of corticosteroid therapy is recommended. Based on the severity of inflammation, clinicians may consider the most potent topical corticosteroid from the locally available options. If necessary, topical therapy may be supplemented with sub-tenon, intravitreal, and systemic corticosteroids. Treatment can be tapered once the inflammation is under control [2]. 

We reported a case of IOI that developed after IVBr for nAMD that had a delay in beginning the treatment because of the lack of reporting a reduction in the vision due to the patient’s cognitive dysfunction. 

## 2. Case Presentation

**Figure 1:**
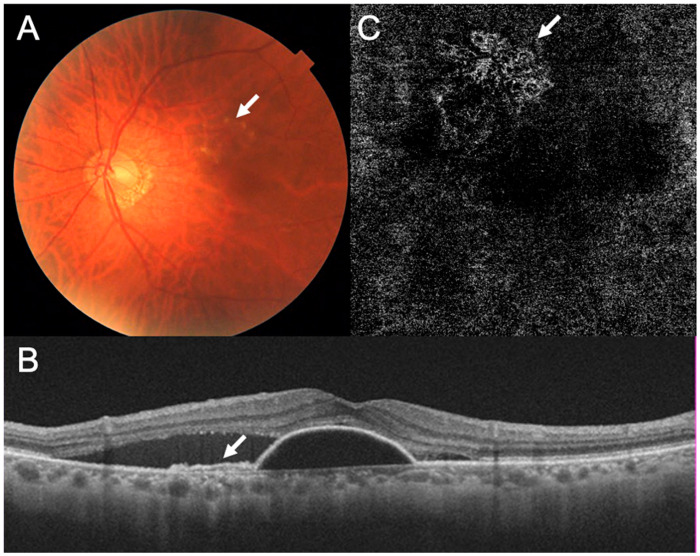
Multimodal imaging findings of the left eye before beginning the anti-vascular endothelial growth factor treatment. (**A**) Fundus photograph showing the slight abnormalities in the retinal pigment epithelium (RPE) (arrow) and hard exudates. (**B**) Optical coherence tomographic (OCT) image showing a double-layer sign indicating type 1 macular neovascularization (MNV) (arrow), serous pigment epithelial detachment, and subretinal fluid (SRF). (**C**) OCT angiography (OCTA) image showing MNV (arrow) in areas corresponding to the RPE abnormalities in the fundus photograph.

A 79-year-old Japanese man was referred to our hospital with a complaint of visual disturbances in both eyes. His decimal best-corrected visual acuity (BCVA) was 0.9 in his right eye and 1.0 in his left eye. The intraocular pressure (IOP) was 13 mmHg in both eyes. Slit-lamp examinations showed no abnormalities except for mild cataracts in both eyes. Ophthalmoscopy showed mild abnormalities in the retinal pigment epithelium, but the presence of hard exudates and serous retinal detachment in his left eye (Figure 1A). Optical coherence tomography (OCT) showed the double-layer sign indicating that the patient had type 1 macular neovascularization (MNV), serous retinal pigment epithelial detachment (PED), and subretinal fluid (SRF) in his left eye (Figure 1B). Optical coherence tomography angiography (OCTA) showed macular neovascularization (MNV) located in the area corresponding to the area of the RPE abnormalities (Figure 1C). Based on these findings, he was diagnosed with nAMD in the left eye, and we began treatment with intravitreal injections of aflibercept (IVA).

**Figure 2:**
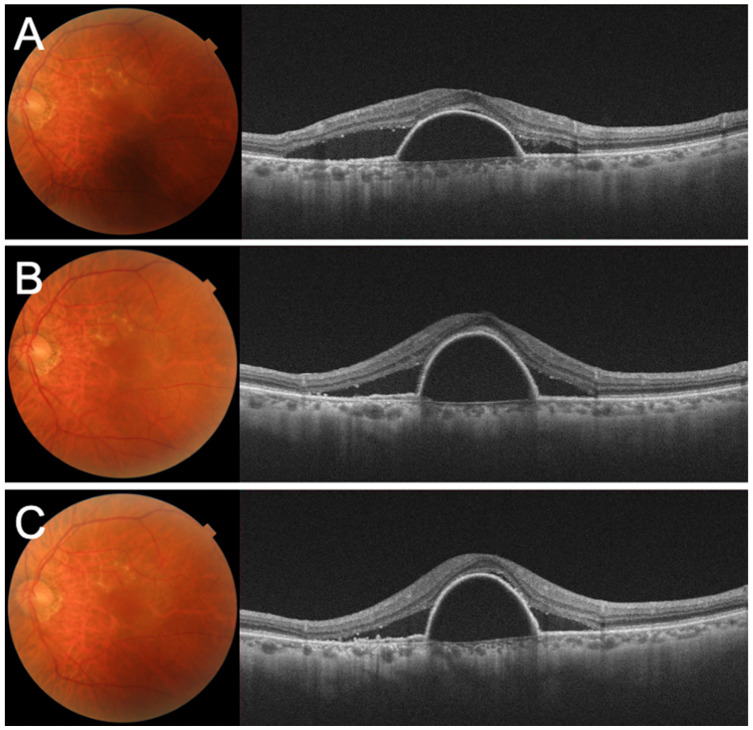
Fundus photographs and optical coherence tomography (OCT) images after the initiation of intravitreal injection of aflibercept (IVA) treatment. (**A**) One month after the first IVA. (**B**) One month after the second IVA. (**C**) One month after the third IVA. The serous pigment epithelial detachment and subretinal fluid is still present.

**Figure 3:**
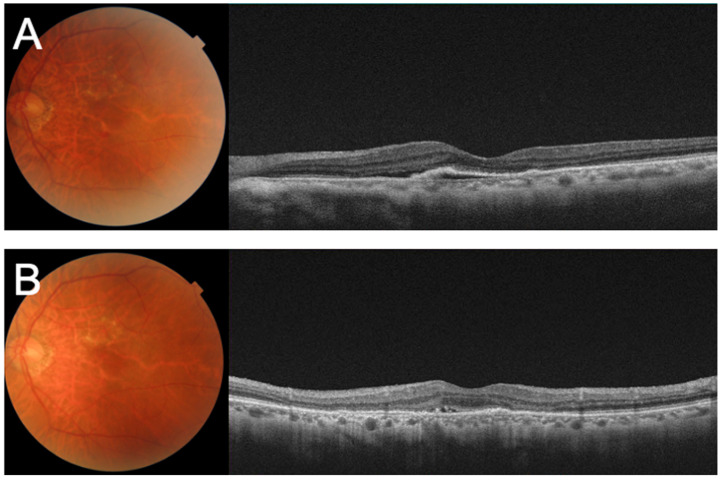
Fundus photographs and optical coherence tomography (OCT) images after switching to intravitreal brolucizumab (IVBr) treatment. (**A**) OCT images one month after the first IVBr showing serous pigment epithelial detachment (PED) and subretinal fluid. (**B**) OCT image one month after the second IVBr showing that the serous PED and SRF reduced.

Despite the three-monthly IVA injections, the serous PED and SRF remained, and the BCVA gradually decreased to 0.1 (Figure 2A–C). We concluded that his left eye was refractory to aflibercept and switched his treatment to brolucizumab. After there monthly IVBr injections, the decimal BCVA improved to 0.3, and OCT showed that the serous PED and SRF had been resolved (Figure 3A,B). He was then instructed to be aware of symptoms associated with IOI. 

Three weeks after his third IVBr injection, he was referred to our hospital with a complaint of decreased vision in his left eye. The reduced vision had been noted two weeks earlier by the patient, but he failed to report it.

**Figure 4:**
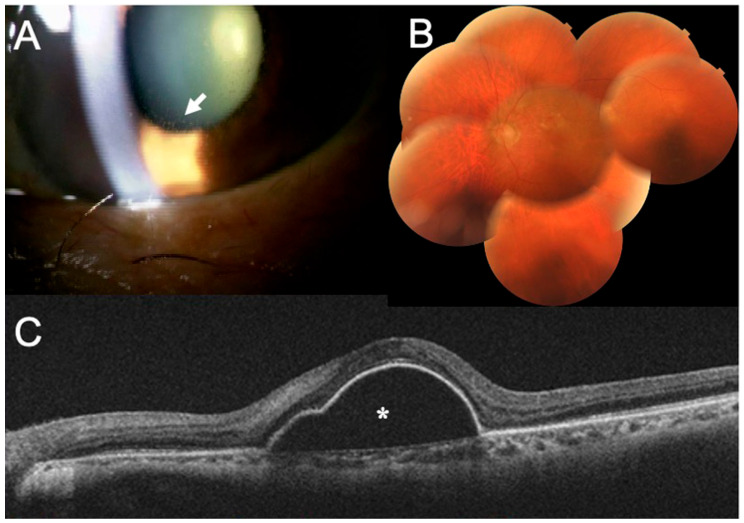
Intraocular inflammation findings three weeks after the third IVBr treatment. (**A**) Slit-lamp image showing keratic precipitates on the posterior surface of the cornea (arrow). The keratic precipitates are depicted as small spots with no clear outline as seen in transillumination. (**B**) Fundus photographs showing the absence of vasculitis. (**C**) Optical coherence tomography (OCT) image showing the recurrence of the serous pigment epithelial detachment (asterisk).

Our examination showed that his BCVA had decreased to 0.02, and the IOP increased to 43 mmHg in his left eye. Slit-lamp examination showed keratic precipitates in his left eye (Figure 4A), and ophthalmoscopy showed an absence of retinal vasculitis in his left eye (Figure 4B). However, OCT revealed the recurrence of the serous PED (Figure 4C). 

**Figure 5:**
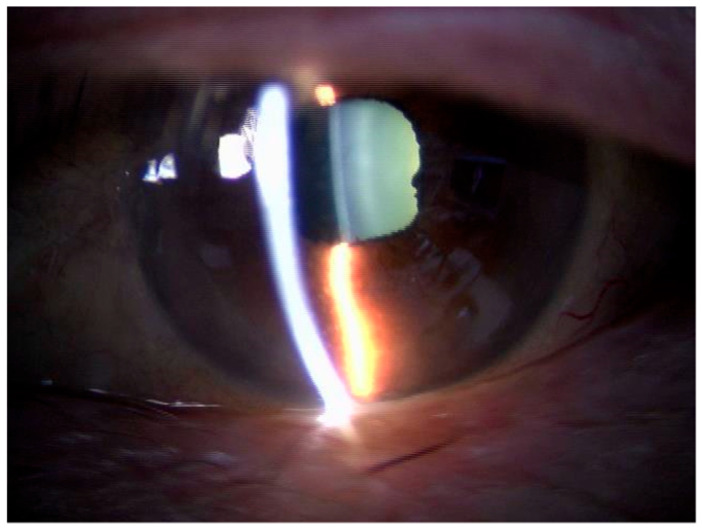
The slit-lamp findings two weeks after the initiation of treatments for intraocular inflammation. Slit-lamp examination showing that the keratic precipitates are not present.

Because the left eye showed signs of IOI mainly in the anterior chamber, we began treating the IOI and ocular hypertension with an intravenous infusion of D-mannitol, 250 mg of oral acetazolamide 2/day supplemented with topical brinzolamide, timolol M-maleate, betamethasone, and latanoprost. The IOP normalized two weeks later, but his symptoms were unchanged with the BCVA in the left eye remaining at 0.02. However, the IOP was normal and no keratic precipitates were visible (Figure 5). These treatments improved the inflammation, but the BCVA remained reduced. We suggest that the lack of a complete recovery of the BCVA was caused by both the recurrence of serous PED and the delay in treating the IOI. 

Because we remembered that we had to explain the cause of his symptoms and the importance of early treatments repeatedly, we suspected that the patient had some degree of cognitive dysfunction. To assess his cognitive functionality quantitatively, the Mini-Mental State Examination (MMSE) was performed. A score of 27 points or higher is normal for this examination. His MMSE score was 20, and he was classified as highly suspicious of having dementia. 

He did not wish to continue with additional treatments for his nAMD, so we followed him without any treatment. 

## 3. Discussion

Brolucizumab is an anti-VEGF agent that was launched in 2019 for the treatment of nAMD. Brolucizumab was reported to be well tolerated; however, the incidence of IOI was higher with brolucizumab than with aflibercept. In 2020, the American Society of Retina Specialists circulated safety updates reporting on the development of retinal vasculitis (RV) in patients treated with brolucizumab. Some of these cases were reported to develop occlusive RV with an associated reduction in vision. The post-marketing report stated that the rate of RV and/or retinal vascular occlusion (RVO) was approximately 15.1 per 10,000 injections [3]. It was also reported that severe vision reduction could develop secondary to retinal arteriolar occlusions after multiple IVBr injections [4]. Therefore, retinal specialists have emphasized the need for early diagnosis and for prompt and appropriate treatment of the IOI to minimize the risk of the progression of these adverse events [5]. For inflammation confined to the anterior chamber, frequent administration of potent topical corticosteroids may be adequate to control the inflammation. If a posterior segment inflammation is detected or even suspected, intravitreal, sub-tenon, or systemic corticosteroids should be administered [3].

Anti-VEGF injections for nAMD have other serious ocular side-effects including endophthalmitis, retinal detachment, and vitreous hemorrhage. Therefore, patients are usually well-informed on the treatment protocols, and consent must be obtained before the first dose is injected. However, before initiating or switching to brolucizumab, the risk of an RO and vision reduction should be explained to the patient more carefully than at the start of anti-VEGF therapy. In addition, patients should be informed of the signs and symptoms associated with IOI, and the importance of immediately returning to the clinic if such signs and symptoms develop [3]. However, not all nAMD patients comprehend the explanations about the treatment protocol or complications.

A meta-analysis combining both unadjusted and adjusted outcomes showed that dementia and Alzheimer’s disease (AD) were significantly associated with an increased risk of AMD [6]. In the same way, patients with AMD were significantly associated with the development of AD or senile dementia compared with the controls [7]. Johnson et al. first identified amyloid β-peptide (Aβ), the major proinflammatory component of senile plaques in AD, in the drusen of eyes with AMD. This indicated that amyloid β-peptide may play a fundamental role in the activation of complement-mediated events during drusen formation [7]. Similar to its role in AD, Aβ in the drusen may lead to the progression of AD by causing oxidative stress, uncontrolled inflammation, and imbalanced angiogenesis. Such commonly shared pathways in AMD and AD could explain the association of AMD and AD. A previous meta-analysis has indicated that vision impairment is associated with an increased risk of dementia. The lack of physical and social activities in the visually impaired elderly may predispose them to cognitive decline [8]. 

Several methods have been used to assess the relationship between dementia and AMD. The mini-mental state examination (MMSE) is the most widely used short cognitive screening test because it is quick and easy to administer in a clinical setting. The MMSE evaluates cognitive function by scoring the degree of memory retained, calculations, languages, and disorientation based on the answers to 11 questions. The maximum MMSE score is 30 points, and patients scoring less than 26 are classified as having mild cognitive impairment, and those scoring less than 21 are classified as having suspected dementia. Rong et al. compared the cognitive function scores between AMD patients and control subjects using various examinations including MMSE [6]. The results showed that patients with AMD had significantly lower MMSE summary scores than the controls, suggesting the possibility of subclinical dementia. In our case, we diagnosed his left eye as refractory to aflibercept and switched the treatment to brolucizumab. Before starting the treatment, we explained the complications and treatment schedule of the anti-VEGF therapy. Since the patient, like other nAMD patients, did not question our explanations, we assumed that he fully understood the anti-VEGF treatment. In fact, he was treated with aflibercept according to the plan.

Before starting the brolucizumab, we carefully explained to him the risk of IOI and vision loss after IVBr. We informed the patient on the symptoms associated with IOI after IVBr and the importance of immediately returning to the clinic if he experienced such symptoms. Unfortunately, no family members were present at the time of the explanation. Although it appeared that he understood our explanation well, he came to our clinic two weeks after he became aware of a reduction in vision in his left eye. Although the inflammation improved with the topical steroids, initiating treatment for the IOI required time, and the vision did not recover to earlier levels. We suspected that he did not return because of the patient’s cognitive impairment as demonstrated by his MMSE score of 20. 

Our findings demonstrated the importance of determining the cognitive function of patients with suspected cognitive reduction before starting treatment and explain the sign and symptoms of IOI to the family members as well as the patient. For patients with suspected dementia, it has been considered that more frequent follow-up visits should be made to detect the IOI and RO as early as possible. In addition, to reduce the development of IOI following IVBr, prophylactic sub-tenon injections of triamcinolone acetonide (STTA) should also be considered [1].

## 4. Conclusions

We conclude that when prescribing IVBr treatment, the cognitive capabilities of the patient should be determined, and a close monitoring of the eye should be performed for the early detection of the IOI in patients with cognitive impairment or suspected cognitive impairment. Because nAMD is significantly associated with dementia, clinicians need to explain the symptoms of IOI to both the patient and family members, and to plan additional follow-up visits more frequently. In addition, combination therapy with STTA and IVBr should be considered if necessary. Clinicians should also be aware that some patients may have latent cognitive decline even without findings suggesting cognitive dysfunction.

## Data Availability

All data are included in the manuscript.
